# A Treatise on Aphasia and Other Speech Defects

**Published:** 1899-09

**Authors:** 


**A Treatise on Aphasia and other Speech Defects.** By H. Charlton Bastian, M.D., F.R.S. Pp. viii., 366. London: H. K. Lewis. 1898.

Dr. Bastian is so high an authority on aphasia that this book, which gives a complete and authoritative exposition of his views on the subject, will be read with great interest. The remembrance of the admirable Lumleian lectures of 1897, dealing with "some problems in connection with aphasia and other speech defects," will arouse favourable anticipations with regard to the present volume, which are not disappointed in it. Dr. Bastian is well-known for the lucid manner in which he presents to his readers some of the most intricate problems with which medical men have to deal. He has wisely in this book illustrated his observations with reports of typical cases, well chosen, and for the most part completed by a necropsy. The careful study of such reports gives great help in the actual working out of the problems involved in cases of aphasia, and brings out more clearly the basis of fact on which the theories of speech-defect are founded. The book is also free from the tendency found in some writers on aphasia to construct elaborate classifications, which have very little to support them in the way of proved pathological or psychological data. We are grateful to the author for the simple nomenclature which he adopts, and which is easily understood.

Besides expressing his own views as to the nature and origin of aphasic defects, Dr. Bastian gives throughout the book a very fair presentment of the views of other writers, especially on the points in which they differ from him. It would be impossible within the limits of this notice to enter into any discussion of the many vexed questions which beset the subject. Dr. Bastian attaches special importance and brings forward a number of cogent arguments for the activity of the word-centres in the right as well as in the left hemisphere, and for the compensatory action of the former in disease affecting the latter; this possibility, first advanced as a probable explanation of the preservation of slight or disordered speech after destruction of the left auditory word-centre, was originally due to him. But for this and the many other difficult and interesting points connected with the disorders of speech we must refer the reader to the book itself, in which he will find that they receive a full and adequate discussion.

The work can be confidently recommended as a trustworthy guide by the hand of a master. The excellent introductory chapters deserve special mention; and the final chapters on etiology, diagnosis, prognosis, and treatment of speech-defects are particularly good, and will be found of much value in practice. Where necessary the text is illustrated by clear diagrams, and a carefully thought-out scheme for the examination of patients is given.

